# Case Report: Adolescent With Autism and Gender Dysphoria

**DOI:** 10.3389/fpsyt.2021.671448

**Published:** 2021-05-26

**Authors:** Sanja Zupanič, Ivona Kruljac, Mojca Šoštarič Zvonar, Maja Drobnič Radobuljac

**Affiliations:** ^1^Center for Mental Health, University Psychiatric Clinic Ljubljana, Ljubljana, Slovenia; ^2^School of Medicine, University of Ljubljana, Ljubljana, Slovenia

**Keywords:** autism spectrum disorder, transgender (LGBT), gender dysphoria, case report, child and adolescent, depression, anxiety

## Abstract

There is increasing clinical evidence of an association between gender variability, gender dysphoria (GD), and autism spectrum disorder (ASD). This seems to be a two-way relationship, a person with GD is more likely to be diagnosed with ASD and vice versa. In youth, it is important to distinguish whether the presented symptoms are a manifestation of ASD focus on special interests or symptoms of co-occurring GD. This distinction is crucial in the process of planning reversible and especially irreversible medical procedures in the context of treatment. We present the case of a birth-assigned female adolescent with GD, who enrolled in our clinic at the age of 16.5 years with “being transgender” as her main complaint accompanied by a wish for surgical breast removal. His (as the patient prefers to use male pronouns) medical and developmental history involved obesity, hyperlipidemia, delays in social and language development and specific interests and rituals. He presented with half a year of untreated depression, suicidal thoughts and non-suicidal self-injuring, social phobia and relative social isolation. Comprehensive clinical assessments revealed a female karyotype (46, XX), normal female genitalia and unremarkable hormonal status. Clinical psychological assessments reported GD, ASD with average intellectual abilities and co-occurring symptoms of depression and anxiety. Other disorders, such as psychosis, personality disorder and dysmorphophobia, were excluded during longer-term diagnostic and psychotherapeutic processes. Our first aim was to build a good therapeutic alliance with the patient and treat depression and suicidality. He refused to take sertraline, but took a St. John's Wort over-the-counter peroral preparation in the form of infusions. His mood improved, he was no longer suicidal and started social transitioning, yet he remained socially phobic. At the time of writing, he is 20 years old, waiting for bilateral mastectomy and receiving regular triptorelin depot and testosterone depot intramuscular injections. Even though the diagnostic procedures and transition process in autistic gender diverse adolescents may take longer than in non-autistic individuals, ASD is not a contraindication to the gender transition process. We present a well-documented case of a slow social and medical transition resulting in gradual improvement of co-occurring symptoms of GD.

## Introduction

Gender dysphoria (GD) is defined by a persistent incongruence between one's assigned sex at birth and one's experienced gender (1).

Clinical research showing evidence of an association between GD and autism spectrum disorder (ASD) is rising (2, 3). In a 2010 Dutch study the reported incidence of ASD in GD children and adolescents was 7.8% (4). Further studies suggested the presence of gender variance in 3.8–7.2% of persons with ASD, while autistic symptoms or diagnosis were present in 5.5–21.3% of people with gender dysphoria, more commonly in birth-assigned females (3). This seems to be a two-way relationship, meaning that a person with GD is more likely to be diagnosed with ASD (compared to the general population) and vice versa, someone with ASD is more likely to experience GD (5). It has also been noted that GD and ASD sometimes co-occur in atypical presentations, which makes a correct diagnosis and treatment options for GD even more difficult (3).

Research has shown an association between elevated fetal testosterone and autistic traits (6). Children diagnosed with ASD, especially girls, are more likely to have elevated androgen levels (7) and an increased incidence of associated disorders (8). Elevated testosterone levels could explain the occurrence of GD in birth-assigned females with ASD, but not in birth-assigned males with ASD.

There is an assumption that the brains of males and females differ regarding the predominance of empathizing or systemizing (9). In the general population empathizing predominates in women while systemizing predominates in men. Problems with empathizing in birth assigned females with ASD could lead to difficulties in interacting with other females who use empathy as the predominant mode of interaction and facilitate identification with males in whom the predominant brain function is systemizing (10). Due to their diminished capacity for empathy, it is theorized that individuals with ASD are less likely to be preoccupied with social norms and prejudices and, as a result, have less difficulty expressing gender diversity (11).

Another possible link between GD and ASD could be the presence of intense, obsessive interests on a gender-specific theme (12). The distinction between an obsession with gender issues as a symptom of ASD and GD co-occurring with ASD is of paramount importance in the process of planning reversible and especially irreversible medical procedures in the context of GD treatment (13).

According to the Standards of Care (SoC) of the World Professional Association for Transgender Health (WPATH), the assessment and treatment of GD should be multidisciplinary, longitudinal, and individualized. Mental health professionals working with GD children and adolescents should directly assess GD, provide family counseling and supportive psychotherapy to assist children and adolescents with exploring their gender identity, alleviating distress related to their GD and ameliorating any other psychosocial difficulties, assess, and treat any coexisting mental health concerns and refer adolescents for additional physical interventions if indicated. They should also provide children, youth and their families with information and referral for peer support (14). It has been pointed out that due to the high vulnerability of the gender diverse population of minors the main rule that should guide decision-making during assessment and treatment procedures is prudence (15).

Due to the assessment difficulties and from fear of making an incorrect diagnosis that would result in irreparable damage given the irreversibility of medical sex-reassignment procedures, many professionals are reluctant to treat children and adolescents with GD or wish to postpone their treatment (16). This is even more pronounced in adolescents with comorbid ASD, as the co-occurrence presents significant diagnostic and treatment challenges given the social, adaptive, self-awareness, communication, and executive function complexities of youth with ASD (13). In 2018 Strang et al. suggested the following initial clinical guidelines for adolescents with co-occurring ASD and GD: emergency hospitalizations in extreme cases to prevent self-harm/mutilation and concurrent assessment of ASD and gender-related issues. They offered an assessment protocol, suggesting assessment of cognitive skills, communication skills, emotional functioning, social cognition, and capacity for self-advocacy, a structured interview to assess the intensity and pervasiveness of GD and an additional report from outside sources (parents). The protocol is followed by a treatment checklist covering basic psychosocial and medical fields, such as putting together the clinical team, providing psychoeducation, etc., but also suggesting two key clinical questions to be answered before medical transition: (1) Is the GD clear, urgent, pervasive, and persistent over time? (2) Does GD increase or decrease with intervention? If the answer to the first question is affirmative, then GD is not caused by comorbidities or symptoms of ASD (e.g., preoccupations) and if the answer to the second question is that GD is decreasing with gender affirmative treatment, then medical transition is indicated. Issues of risks, benefits and treatment side-effects should be understood by the adolescent and the family (13).

We present a well-documented case of a slow social and medical transition in an adolescent with ASD and GD resulting in gradual improvement of symptoms of GD. The adolescent was treated at the Slovene Child and Adolescent Gender Outpatient Clinic (established in 2015) from 2017 until the time of writing.

## Case Description

### Patient Information

The presented patient is a white Caucasian birth-assigned female, who first visited our clinic at the age of 16.5 years due to GD, depression and suicidal thoughts. At the first meeting, he explained he was a “trans-male” and asked the therapist to use male pronouns. It was his wish for us to use male pronouns in this article as well.

His medical history involved obesity since early childhood and additional weight gain and hyperlipidemia after puberty (at 9 years of age). Menarche occurred at the age of 12. He had been treated for obesity since age 11. He had regular check-ups with a pediatric endocrinologist.

He was born at the 37th week of gestation without complications. Initially he was visiting a developmental outpatient service, where he was receiving speech therapy for developmental language delay and articulation disorders. As a toddler he was described as sensitive, irritable, stubborn, and shy in social interactions. Spontaneous social interactions with his peers have always been scarce. His primary school psychological assessment revealed average but disharmonic intellectual abilities, with slower psychomotor speed, therefore he received educational adjustments during schooling. Occasionally he was bullied at school for being overweight. He reported not feeling connected with his schoolmates and he spent most of his time at school alone. When he was seven, his parents divorced and in this period his mother noticed he had severe nightmares. They consulted a child and adolescent psychiatrist for advice, but he was not provided with regular treatment.

His special interests were fantasy animals and robots. He kept some of his childhood rituals, such as using a stopwatch every morning to measure exactly 5 min before arising from bed. He never liked proverbs or metaphors because he didn't understand them. To relieve his social anxiety and loneliness he pretended to be a part of a TV show several times a day. He described himself as a “tomboy,” always wearing neutral or more masculine clothes. He completed his primary school successfully.

He described his sexual orientation as bisexual, however, he hasn't had any intimate relationships yet. At the age of 15, he met a transsexual person and discovered similarities between the two of them. Half a year later he revealed his feelings of GD to his mother, asked her to use male pronouns and chose his male name. She promised him support, but believed this was just a developmental phase and was not ready to follow his wishes, which made him sad. His GD was particularly focused on his breasts. He didn't mind his regular monthly “bleedings” since they “didn't show.” He couldn't talk about his feelings to his schoolmates, because he couldn't imagine that their reaction would be supportive. He mentioned his transgender feelings to two of his friends, both females, and one of them started using male pronouns and his male name, which positively affected his mood.

After his 16th birthday his mood deteriorated. He reported feelings of depression lasting for several weeks with frequent bouts of crying in the evenings, occasional thoughts of suicide with two episodes of non-suicidal self-injuring by scratching. His social anxiety intensified. He had no history of substance abuse. His biggest wish was mastectomy.

### Family History

His father was diagnosed with a schizotypal personality disorder and suffered from a psychotic episode before the patient's birth. His mother was treated for depression for several years and was recently diagnosed with an early-stage malignant melanoma. A reason for the divorce was his father‘s occasional violence toward his mother. Both parents formed new families, which remained stable. He has non-conflicting relationships with both stepparents, but he is not close to either of them. He has a 9 years younger brother from the mother's second marriage.

### Clinical Findings

At the first appointment, he appeared to be a neat, male-looking, overweight teenager, with short hair, wearing military-colored male clothes. He was alert and oriented. His speech was slow with increased latencies, taking long pauses before answering. His use of language was specific, unusual, he repeatedly mistook the meanings of words and phrases. He avoided using gender defining pronouns and sentence structures, so the sentences were completely without pronouns. His thought process was logical; there was no evidence of hallucinations or delusions. His affect was mildly depressed and blunted, although he was seen holding back tears from time to time. He was anxious, reserved and he described symptoms of social anxiety. He reported suicidal ideations, but was able to agree on an anti-suicide security agreement. He expressed his GD, focusing primarily on his breasts. Some typical characteristics of ASD were evident (difficulties in mutual gaze and in social interactions, language peculiarities, specific interests, rituals), which warranted further evaluation.

### Diagnostic Assessment

Following WPATH SoC (14), we performed psychiatric, clinical genetics, gynecological, pediatric endocrinological, and clinical psychological assessments. The medical data revealed a female karyotype (46, XX), normal female genitalia, unremarkable hormonal status, obesity (body mass index 30 kg/m2) and hyperlipidemia.

The clinical psychological assessment was conducted by two clinical psychology consultants (subspecialized in the fields of gender incongruence and ASD). Following clinical psychological auto- and heteroanamnesic interviews, diagnostic methods were selected to differentiate between GD, ASD, and other potential mental health disorders (such as psychosis, depression, anxiety disorder, and personality disorder). The following tools were successfully utilized: Draw-a-Person Test, Gender Identity Dysphoria Questionnaire for Youth and Adults (GIDYQ-AA), Rorschach test, Thematic Apperception Test, Raven's Standard Progressive Matrices test (SPM), Digit Span Test (from Wechsler Memory Scale), Minnesota Multiphasic Personality Inventory for Adolescents (MMPI-A), Sentence Completion Test, Millon Clinical Multiaxial Inventory (MCMI-II), Autism Diagnostic Interview (ADI-R), Diagnostic Interview for ADHD in Adults (DIVA), Self-Concept Questionnaire—Form A (AFA), Bender Gestalt Test (BGT), Ritvo Autism Asperger's Diagnostic Scale—Revised (RADDS-R), and the Autism Diagnostic Observation Schedule (ADOS). The results are presented in Table 1.

**Table 1:** The results of the clinical psychological assessment.

**Intellectual and cognitive abilities:** The results revealed intact visual-motor performance (BGT), average attention span and working memory, low intellectual abilities (25th percentile) with disharmonic profiles of subtests due to inconsistent mental effort and therefore lower reliability of the results. Performance was better on more difficult subtests suggesting specific concentration problems and the importance of motivational factors (SPM).
**Assessment of ASD and ADHD:** The results on the RADDS-R were above the cut-off score suggesting the possibility of ASD, which was later confirmed by the ADI-R and then by ADOS classification (Asperger syndrome). Problems with communication (inability to understand metaphoric speech or using idiosyncratic speech), which was less spontaneous and only partially accompanied by non-verbal signs (eye-contact, gesticulation), were evident. Problems with understanding social situations and mentalizing about himself and others were also present. At the assessment with the client's mother, ADI-R and DIVA were also given. In the latter, the adult subsections were skipped and only the child and adolescent subsections were performed in order to exclude attention deficit and hyperactivity disorder and gather more information about ASD and the adolescent's broader functioning.
**Gender dysphoria assessment:** GD was confirmed by in-depth diagnostic interviews accompanied by the GIDYQ-AA questionnaire (raw score = 63, *M* = 2.3), which was within the range typical for adolescents with GD whose birth-assigned sex was female.
**Emotional functioning:** Subsequent testing revealed increased levels of anxiety and depression symptoms (MMPI-A and MCMI-II T-scores on these subscales were over 70). He also had self-esteem deficits (AFA score was within the 1st percentile) which were evident on all subscales: family (10th percentile), body-image (4th percentile), emotional and social (both lower than 1st percentile), while his academic auto-concept was elevated (85th percentile), probably due to unrealistic self-perceptions.
**Personality assessment:** Projective techniques (Rorschach test, scored with Rorschach Assistance Program (Ver. 3) by Virtual Psychology) revealed further information about the client's functioning. On the surface he was functioning quite well (Adj D = +1), yet he seemed to be having problems with emotion regulation control (CF + C = 5, FC = 3, Afr = 0.87, a: *p* = 5: 2) and perception and thinking (PTI = 5, XA% = 0.5, X-% = 0.43, SumLv2 = 4, FAB2 = 3, *R* = 28, WSUM6 = 29, *M*- = 2). He seemed to be guided more by impulses than rational thinking (EB = 3: 7), which occasionally manifested in weakened impulse control (resulting in excessive food intake, for example). His reality control capacities were weak, there were indices of a schizoid withdrawal into isolation as well as the presence of hypervigilance (confirmed by HVI index). He described vivid de-realizations, occurring mainly in social situations (social anxiety in terms of ASD). Comprehensive qualitative evaluation of Rorschach test, TAT and Draw-a-Person Test revealed problems with identity differentiation and weak identity structure. Concrete thinking (Blends: *R* = 2: 28) and fragile self (Fr + rF = 2) with a very limited capacity for mentalization [Pure H = 1, (H) = 7, SumT = 0, GHR: PHR = 2: 7] were also features shaping his identity perception and identity expression. Due to his young age (17 years) we could not diagnose personality disorder, yet the results on MCMI-II Schizotypal subscale were increased (BR = 113) and suggested caution in further treatment and medical interventions.

*BGT, Bender Gestalt Test (BGT); SPM, Raven's Standard Progressive Matrices test; RADDS-R, Ritvo Autism Aspergers Diagnostic Scale – Revised; ADI-R, Autism Diagnostic Interview; ADOS, Autism Diagnostic Observation Schedule; DIVA, The Diagnostic Interview for ADHD in Adults; GIDYQ-AA, Gender Identity Dysphoria Questionnaire for Youth and Adults; MMPI-A, Minnesota Multiphasic Personality Inventory for Adolescents; MCMI-II, Millon Clinical Multiaxial Inventory; AFA, Self-Concept Questionnaire – Form A; Adj D, Adjusted D score; CF, Color Form responses; C, pure Color responses; FC, Form Color responses; Afr, Affective ratio; a: p, active: passive ration; PTI, Perceptual Thinking Index; XA%, Form Appropriate Extended; X-%, Distorted form; SumLv2, sum of level 2 responses; FAB2, number of level 2 fabulations; R, number of Responses; WSUM6, weighted sum of 6 special scores; M-, distorted human movement responses; EB, Erlebnistypus; Fr + rF, Form-reflection responses; Pure H, pure Human responses, (H), fictional Human responses; SumT, sum of Texture responses; GHR: PHR, Good Human Responses: Poor Human Responses ratio; BR, Base Rate score*.

The main diagnostic dilemma was whether the wish for mastectomy could be an obsessive symptom within ASD and remained even after the completion of a comprehensive clinical psychological evaluation, suggesting atypical presentation of adolescent co-occurring GD and ASD. An individualized watchful waiting approach in combination with regular psychiatric check-ups was suggested. A supportive psychotherapeutic approach seemed to be more appropriate than an affirmative one, since the former provides more space for gender exploration and the possibility of different gender outcomes.

### Therapeutic Intervention, Follow-Up, and Outcomes

Our first goal during the assessment phase was to build a good therapeutic collaboration and treat his depression and suicidality. He refused to take sertraline, but he agreed to take a St. John's Wort over-the-counter preparation in the form of infusions taken three times daily. Similar to the antidepressants, he was not interested in hormonal therapy both due to concerns about its effects on his weight and strongly fearing taking “artificial synthetic substances that would affect his entire body.” Due to the difficulties in imagining his peers' thoughts, feelings and reactions, he was initially reluctant toward transitioning socially. For the first half year, he had regular by-weekly and monthly check-ups with a child and adolescent psychiatrist including systemic family therapy sessions with his mother. His father declined any co-operation in his treatment. He was also regularly attending individual supportive psychotherapy sessions with a psychologist. In this period his mood improved, he was no longer suicidal and he asked for less frequent visits. He discontinued his medication with St. John's Wort in spite of the psychiatrist's advice against it.

He did not want to undergo full social transition despite the fact that he was wearing male clothes, he cut his hair short and his parents knowing about his transgender feelings.

At the age of 17 he demanded that his wish for mastectomy be presented to a multidisciplinary medical council, but his request was denied due to his age, ongoing diagnostic dilemmas and a refusal to transition socially. His mother agreed on very gradual gender-affirming procedures as she was uncertain about the permanence of his GD. He refused the offer to join the therapeutic group for transgender adolescents on several occasions; his mother visited the support group for parents only once. He joined peers with a similar experience in a non-governmental organization for transgender people.

In psychotherapy he frequently wondered why his GD peers were able to undergo transition faster than him, but he was unable to mentalize the differences. He was not able to imagine how his peers would have reacted to a person with a mastectomy and a female name. He was still avoiding using pronouns. When introducing himself he waited for the other person to choose how they would address him and never offered corrections or explanations.

At the age of 18 he considered hormonal treatment because his GD intensified during his regular monthly periods. However, due to his worries about the side effects he again declined the offered hormonal blockers.

As his wish for mastectomy persisted and there were no improvements in the symptoms of GD, we presented his case to the multidisciplinary medical council again, where it was decided to consult foreign professionals who have experience with subjects with such comorbidities. Both consulted professionals wrote expert opinions, supporting gender-affirming procedures while keeping the support offered in terms of psychotherapy and child psychiatric counseling. They also suggested adolescent group therapy, which he persistently refused.

At the age of 19 he decided to commence with hormonal treatment. He was administered triptorelin injections 11.25 mg IM every 3 months (an estrogen inhibitor). The experienced side effects (hot flashes, blushing, mood swings) disappeared after the second dosage. He was happy about the cessation of his periods and continued with the medication.

He attended child psychiatric consultations irregularly and skipped three quarters of the offered visits; he ceased attending the psychotherapy sessions. He finished secondary school and commenced with multimedia studies, which, together with his new student role, increased his anxiety. On his psychiatrist's advice he recommenced taking the St. John's Wort preparation.

He received his first dose of testosterone (testosterone undecanoate 1,000 mg IM every 12 weeks) together with triptorelin, reporting no side effects from the prescribed medications. After initial administration of testosterone, he reported a decrease in anxiety and decided to discontinue the St. John's Wort preparation. He changed his name to a gender-neutral name, but refused to officially change his legal gender. His explanation was that this change was too big for him.

**Figure 1:**
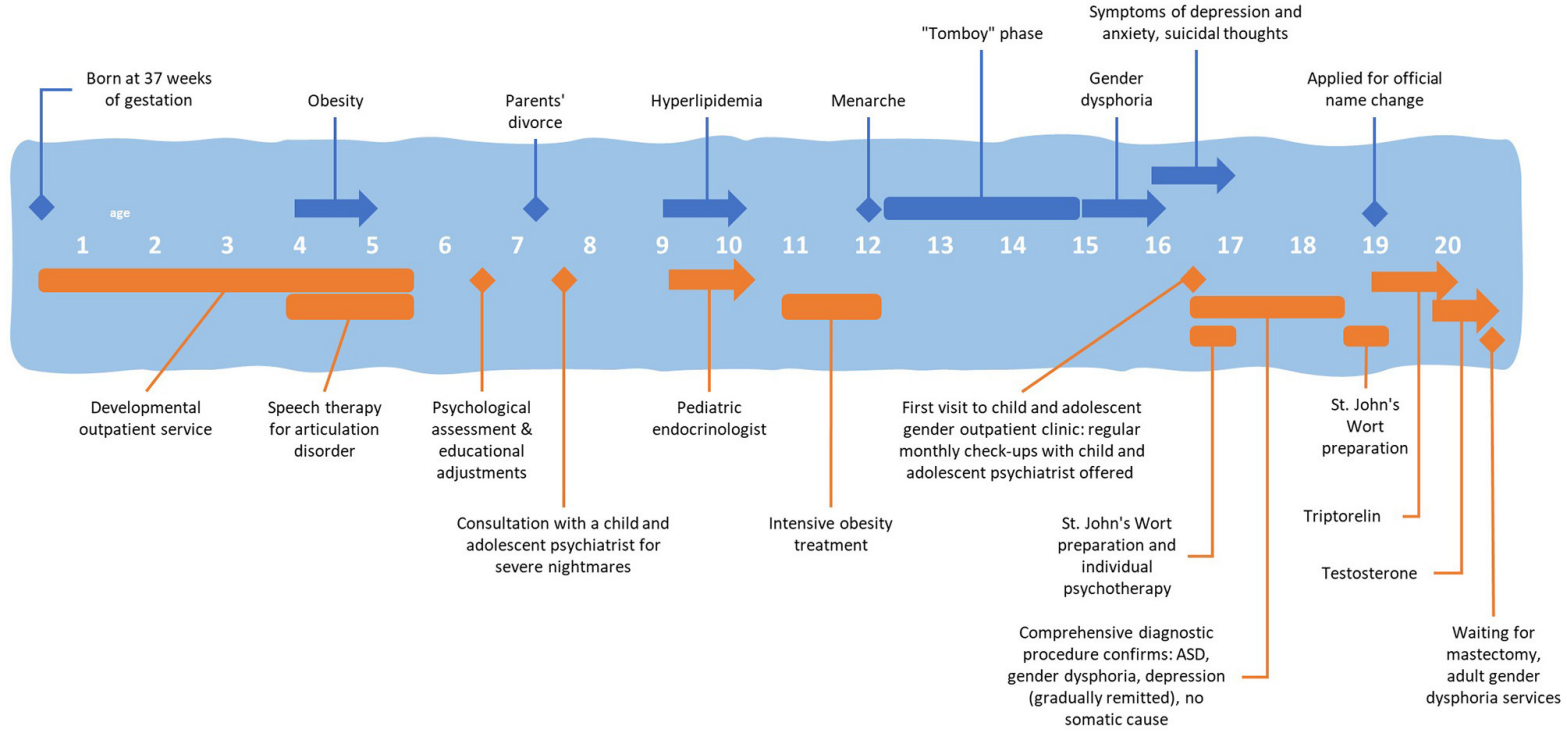
Timeline with relevant data from the continued care of an adolescent patient with comorbid ASD and gender dysphoria in the Slovene Child and Adolescent Gender Outpatient Clinic.

At the time of writing, he is 20 years old and waiting for bilateral mastectomy. His mood is currently euthymic, his symptoms of anxiety and social phobia are under control, partly due to improved self-esteem after receiving hormonal therapy, partly due to social isolation and on-line schooling during the COVID-19 pandemic. He is in the process of transitioning to the adult GD services. The timeline with relevant data from the continued care is shown in Figures Figure 1.

## Discussion

We present a case of a gender dysphoric adolescent with comorbid ASD, the first confirmed comorbid case at the Slovene Child and Adolescent Gender Outpatient Clinic. The timeline of his assessment and treatment, which has paved the way for the management of other youth with similar comorbidities, is shown. The strength of our report is the thorough assessment and multi-disciplinary care, as well as the length of follow-up. The limitation is the inability, due to the small number of ASD patients, to include the patient and the parents in an ASD-only adolescent and parent group therapy, as a recent study advises (17).

The symptoms of the presented case supported the findings of the studies reporting a higher risk of depression, anxiety, suicidal thoughts, attempts, and self-injury in GD adolescents compared to their non-transgender peers (18). The symptoms are reported to be highest in the individuals with both conditions (GD and ASD) (19). Social isolation is additionally increased among those with comorbid GD and ASD (20) as was seen in the presented patient as well.

A protective factor against depression in transgender adolescents is parental support (21). Unfortunately, as seen in this patient, those with a GD-ASD comorbidity often face parental skepticism when reporting gender-related distress, thus intensifying their distress, and hopelessness (21, 22).

Diagnosing GD in presented adolescent with ASD was complex due to ASD-related specifics in communication, self-awareness, and executive function. Concrete thinking, as well as struggles with ambiguity and thinking about the future, made the assessment of his understanding of the long-term and social implications of gender transitioning very difficult as elsewhere reported (13). As in the presented case, assessment and treatment of gender-related issues in youth with ASD often overlap because the insight, flexible thinking, communication, and the capacity for thinking and communicating about their gender develop over time with the help of ASD treatment (13). Therefore, as later advised in the guidelines, we reevaluated gender-related needs as therapy progressed (13). Some adolescents with this comorbidity struggle with treatment compliance, which was also the case in the presented patient where helpful parent participation to aid with the gender exploration process was missing as well. The adolescent was encouraged to explore his gender identity over time before he was considered for any potentially irreversible gender-related medical treatments (13).

As reported in previous case reports (23, 24) the main clinical dilemma persisting after extensive diagnostic procedures was distinguishing between obsessional symptoms of ASD and symptoms of GD. As advised, the dilemma was mainly resolved by the persistence and worsening of GD symptoms throughout the follow-up and the alleviation of some of the dysphoric symptoms with gradual medical transition (13, 25). Similarly to previously reported features of ASD, such as impaired Theory of Mind and mentalization, an intolerance toward ambiguity, and persistent deficits in social communication and interaction, we noted his inability to undergo social transition, enroll in group therapy and undergo other self-perceived rapid changes (13, 25). Therefore, the patient's transition needed to be more gradual and took longer compared to his GD non-ASD peers (13).

An ASD diagnosis should not exclude the potential for medical GD treatments, including puberty suppression and cross-sex hormone intervention. When deciding on medical treatments that may have irreversible effects more caution should be applied, taking into account the difficulties in comprehending the long-term risks of gender-related medical interventions and the complexity of consenting to treatment. It is important for the clinician to develop a specialized consent plan for an adolescent with ASD and GD. When applied, hormone treatment should be started with lower doses and increased more gradually (13). These recommendations were followed in the treatment plan of the presented patient where the introduction of testosterone was preceded by lengthy supportive treatment and the gradual hormone-blocking actions of triptorelin. The biological changes were well-tolerated by the patient resulting in improvements in depression and anxiety symptoms. In the transgender population, treatment with gender-affirming hormones is proven to improve mental health, including symptoms of depression and anxiety (26). The majority of reported studies found that testosterone improved mood and reduced anxiety in men, women and animal models. The neurobiological mechanisms underlying these physiological effects remain poorly understood (27).

Access to health care can be challenging for individuals with ASD and GD and their assessment and treatment lengthy. However, respecting diversity when providing care to all individuals is very important. Clinical services for such individuals require considerable awareness and sensitive adjustment throughout the care pathway to avoid further marginalization, or oppression and to consider their specific needs (22, 28). We aimed for this goal in the care of the presented patient.

## Conclusions

Autistic gender diverse adolescents require special care and clinical knowledge during the gender-affirming process. The diagnostic process may take longer due to extended and repeated assessments, as well as specific patient and family characteristics. Although ASD itself is not a contraindication to starting the gender transition process, caution, client-centered care, and collaborative decision-making are recommended, as well as regular coordination within the team of professionals involved in the treatment (13). Due to the diversity of our interdisciplinary team and the smallness of our country, we were able to provide such care and provide an opportunity for the patient's improvement even though at the time of enrolment there were no guidelines for the clinical care of subjects with these comorbidities.

## Patient Perspective

Our patient felt that the diagnostic and gender reassignment procedures were too gradual and not as fast as with other GD adolescents. However, although encouraged, he was not able to undergo the procedures any faster. He believed that his main wish (for mastectomy) was not being heard by his parents, his therapist and the medical council for a long time. Meanwhile, the therapist encouraged him to undergo a social transition and to engage in social interactions with his peers, which he didn't feel were his primary goals. After encouragement, he was satisfied with inclusion in a small peer group and with the two important outcomes of the hormonal therapy: a decrease in anxiety and improvement in self-esteem.

## Data Availability Statement

The raw data supporting the conclusions of this article will be made available by the authors, without undue reservation.

## Ethics Statement

Written informed consent was obtained from the individual(s) for the publication of any potentially identifiable images or data included in this article.

## Author Contributions

SZ and IK drafted the manuscript. MŠ and MD assessed and treated the patient and worked with the family. MD contacted the patient for approval and coordinated the approval of the final draft. All the authors contributed to the final version and approved the submission.

## Conflict of Interest

The authors declare that the research was conducted in the absence of any commercial or financial relationships that could be construed as a potential conflict of interest.

## Abbreviations

GD: gender dysphoria
ASD: autism spectrum disorder
SoC: Standards of Care
WPATH: World Professional Association for Transgender Health
GIDYQ-AA: Gender Identity Dysphoria Questionnaire for Youth and Adults
SPM: Raven's Standard Progressive Matrices test
MMPI: Minnesota Multiphasic Personality Inventory for Adolescents
MCMI-II: Millon Clinical Multiaxial Inventory
ADI-R: Autism Diagnostic Interview
DIVA: The Diagnostic Interview for ADHD in Adults
AFA: Self-Concept Questionnaire - Form A
BGT: Bender Gestalt Test (BGT)
RADDS-R: Ritvo Autism Aspergers Diagnostic Scale - Revised
ADOS: Autism Diagnostic Observation Schedule.
